# How to prepare stool banks for an appropriate response to the ongoing COVID-19 pandemic: Experiences in the Netherlands and a retrospective comparative cohort study for faecal microbiota transplantation

**DOI:** 10.1371/journal.pone.0265426

**Published:** 2022-03-17

**Authors:** Bas Groenewegen, Emilie van Lingen, Rogier E. Ooijevaar, Els Wessels, Mariet C. W. Feltkamp, Eline Boeije-Koppenol, Hein W. Verspaget, Ed J. Kuijper, Joffrey van Prehn, Josbert J. Keller, Elisabeth M. Terveer, on behalf of the Study Group of the Netherlands Donor Feces Bank

**Affiliations:** 1 Department of Medical Microbiology, Leiden University Medical Center (LUMC), Leiden, The Netherlands; 2 Department of Gastroenterology and Hepatology, LUMC, Leiden, The Netherlands; 3 Department of Gastroenterology and Hepatology, Amsterdam University Medical Centers, VU University Medical Center, Amsterdam, The Netherlands; 4 Department of Biobanking, LUMC, Leiden, The Netherlands; 5 Reference Laboratory for *C*. *difficile*, LUMC and RIVM (Center for Infectious Disease Control, National Institute for Public Health and the Environment), Bilthoven, The Netherlands; 6 Department of Gastroenterology, Haaglanden Medical Center, Den Haag, The Netherlands; INRAE, FRANCE

## Abstract

**Background:**

Faecal microbiota transplantation (FMT) is an efficacious treatment for patients with recurrent *Clostridioides difficile* infections (rCDI). Stool banks facilitate FMT by providing screened faecal suspensions from highly selected healthy donors. Due to the ongoing coronavirus disease 2019 (COVID-19) pandemic and the potential risk of SARS coronavirus-2 (SARS-CoV-2) transmission via FMT, many stool banks were forced to temporarily halt and adjust donor activities.

**Goal:**

The evaluation of a strategy to effectively continue stool banking activities during the ongoing COVID-19 pandemic.

**Study:**

To restart our stool banking activities after an initial halt, we implemented periodic SARS-CoV-2 screening in donor faeces and serum, and frequent donor assessment for COVID-19 related symptoms. FMT donor and recipient data obtained before (2016–2019) and during the COVID-19 pandemic (March 2020-August 2021) were compared to assess stool banking efficacy.

**Results:**

Two out of ten donors developed COVID-19. No differences during versus before the COVID-19 pandemic were observed in the number of approved faeces donations (14 vs 22/month, *p* = 0.06), FMT requests for rCDI (3.9 vs 4.3/month, *p* = 0.6); rCDI patients eligible for FMT (80.6% vs 73.3%, *p* = 0.2); rCDI cure rate (90.3% vs 89.2%, *p* = 0.9); CDI-free survival (*p* = 0.7); the number of non-rCDI patients treated with FMT (0.5/month vs 0.4/month), and the number of possibly FMT related adverse events (9.5% vs 7.8%, *p* = 0.7). Two FMTs for rCDI were delayed due to COVID-19.

**Conclusions:**

There is a continued need for FMT treatment of rCDI during the COVID-19 pandemic. Appropriate donor screening and SARS-CoV-2 infection prevention measures can be implemented in existing protocols without increasing the burden for donors, and allow safe, effective and efficient FMT during the ongoing COVID-19 pandemic. Stool banks should evaluate their SARS-CoV-2 donor screening protocols for long-term sustainability and efficacy, and share their experiences to help the utilisation, standardisation and improvement of stool banks worldwide.

## 1. Introduction

The Gram positive, spore producing bacterium *Clostridioides difficile* is capable of colonisation and infection of the human gut. In healthy individuals, the immune system along with a complex interplay of the gut microbiota, by competition for food and space, and excretion of metabolites and bacteriocins, suppresses the growth of *C*. *difficile*. This defence mechanism is also known as colonisation resistance [1]. When dysbiosis of the microbiota occurs, typically caused by treatment with antibiotics, *C*. *difficile* can grow out to pathogenic levels and induce diarrhoeal disease, varying from self-limiting and mild diarrhoea to life-threatening pseudomembranous colitis [2]. *C*. *difficile* infection (CDI) is usually treated with the antibiotics vancomycin or fidaxomicin, but relapses occur frequently (15–25%), and relapse rate increases in patients with recurrent CDI. Consequently, a subset of patients suffers from multiple relapses of CDI [2, 3].

In those patients with relapsing *C*. *difficile* infection (rCDI), restoring the perturbed microbiota with faecal microbiota transplantation (FMT), derived from a healthy donor, has proven to be an efficacious treatment, and is currently standard of care [3–7]. Additionally, FMT as new treatment modality in disorders in which a perturbed gut microbiota appears implicated in the pathophysiology, such as ulcerative colitis or Graft-versus-Host Disease, shows promising preliminary results [8].

Stool banks are essential for safe and effective application of FMT, as they enable the availability of thoroughly screened faecal suspensions obtained from healthy donors [9, 10]. However, the ongoing coronavirus disease 2019 (COVID-19) pandemic, caused by SARS coronavirus 2 (SARS-CoV-2), induced a potential risk of faecal transmission of SARS-CoV-2 via FMT [11]. Meta-analyses showed that 18% of COVID-19 patients experienced gastrointestinal symptoms, and 48% had detectable SARS-CoV-2 ribonucleic acid (RNA) present in the faeces, which could be present in the stool for a prolonged time after infection (pooled mean 17 days, maximum 126 days) [12, 13]. To avoid the potential risk of SARS-CoV-2 transfer from donor to patient, many stool banks were forced to halt donor activities, and sometimes transplantation activities as well [14, 15].

In the Netherlands, the first case of COVID-19 was diagnosed on February the 27^th^ 2020, and since then multiple epidemic waves were met (March-May 2020, October 2020-February 2021, February-June 2021), upon which the Dutch government implemented national lockdown measures [16, 17]. The presence of SARS-CoV-2 in the Netherlands increased the urgency for the Netherlands Donor Feces Bank (NDFB) to implement safety measures and donor screening for SARS-CoV-2 to prevent potential faecal transfer of SARS-CoV-2 by FMT.

The global spread of COVID-19 and the potential risk of SARS-CoV-2 co-transplantation with FMT led to the release of a safety alert by the United States Food and Drug Administration (FDA), stating that only stool donated before December the 1^st^ 2019 should be used for FMT until validated SARS-CoV-2 testing of donors and donor faeces was available [18]. In the meantime, FMT experts described how stool banks and FMT facilities could adapt their donor screening procedure and workflow to prevent SARS-CoV-2 cross-transmission from donor to patient [11, 19]. By adhering to these guidelines and adapting their existing workflow and donor screening protocol, most stool banks have been able to resume donor activities in a relatively short time period, while others experienced more difficulties [14, 15, 20].

The application of guidelines by stool banks to their existing protocols induced variation in approaches to prevent SARS-CoV-2 co-transplantation with FMT. Here we describe how the activities of the NDFB were affected by the COVID-19 pandemic, which additional measures and screening were implemented to continue services, and their effect on stool banking and FMT outcomes in comparison with the pre-COVID-19 era. We focus on assuring safe FMT, but on the other hand aim to minimise the screening burden for donors. Since SARS-CoV-2 has become endemic, we share our experiences to allow other stool banks to improve and continue their activities and to treat patients with FMT.

## 2. Materials and methods

### 2.1. Design and population

This is a retrospective comparative cohort study of all activities of a national stool bank before versus during the COVID-19 pandemic. The NDFB is situated at the Leiden University Medical Center. Data was included from all active faeces donors and patients receiving FMT in the daily patient care (rCDI), a clinical trial, or receiving FMT for indications other than rCDI, between March 2020 and August 2021. Patients treated for rCDI were routinely followed to assess possible adverse events, rCDI relapses and development of COVID-19. Follow-up data until October the 25^th^ 2021 was included for this patient group. Data regarding donations made by donors and FMT for rCDI was compared to data from the four years before the COVID-19 pandemic (2016–2019) [7]. Data regarding patients treated for indications other than rCDI was compared to NDFB results obtained in 2019 (January 2019-January 2020).

### 2.2. Response to COVID-19: Additional screening measures for stool donors

Due to many uncertainties on possible transmission routes of COVID-19, the NDFB halted all donor activities from March 2020 to June 2020. Donor activity was resumed in June 2020 after adjustment of the donor screening protocols.

#### 2.2.1. Clinical evaluation of SARS CoV-2 complaints

During the first six months of the COVID-19 pandemic in the Netherlands, donors who agreed to continue donations during the COVID-19 pandemic were contacted by an NDFB employee on a weekly basis to inquire about COVID-19 related symptoms. Questions assessing COVID-19 symptoms were added to the existing short questionnaire assessing recent health status, filled in by donors upon every donation [10].

#### 2.2.2. Microbiological screening of SARS CoV-2

Faeces is collected by the donor in a faecal container to prevent environmental contamination, and handed in within two hours at our laboratory. The faeces is processed into ready-to-use faecal suspensions within six hours of defaecation. Glycerol in an end volume of 10% is added as cryoprotectant, and the faecal suspensions are subsequently stored at -80°C and quarantined until approval for use in FMT. Every two to three months, donor faeces and serum are screened for a list of viral, parasitic and bacterial pathogens [7, 10]. Screening for SARS-CoV-2 RNA in faeces and SARS-CoV-2 antibodies in serum was added to this screening procedure and performed within three working days on samples stored at 4°C. Faeces screening for SARS-CoV-2 was performed with an *in-house* SARS-CoV-2 polymerase chain reaction (PCR) test with the envelope (E)-gene as target, based on the assay described by Corman and colleagues [21]. In short, faeces was pre-treated using Precellys Soil grinding SK38 (Bertin technology, Montigny-le-Bretonneux, France) and deoxyribonucleic acid was isolated using MagNA Pure 96 technology (Roche Diagnostics, Penzberg, Germany). The internally controlled reverse transcription-PCR assay targeting the SARS-CoV-2 E-gene was performed with a 25 μl reaction mixture consisting of 6.25 μl TaqMan Fast Virus 1-step mastermix (ThermoFisher), 0.4 μM of each primer, 0.2 μM of each probe and 10 μl of the nucleic acid extracts. Stool was considered positive at quantification cycle <35, based on internal validation assays. Serum screening for SARS-CoV-2 antibodies was performed using the Architect® SARS-CoV-2 IgG or Alinity® SARS-CoV-2 IgG anti SARS-CoV-2 nucleocapsid protein immunoassay, according to instructions by the manufacturer.

In retrospect, all donors active between December 1^st^ 2019 and March 15^th^ 2020 (before the NDFB instated a three-month COVID-19 lockdown for donors) were serologically tested for SARS-CoV-2. Upon a negative serum test result (in combination with absence of symptoms), the donor was considered COVID-19-negative and faecal suspensions prior to March 15^th^ 2020 were considered safe for FMT after a completed cycle of bookend screening.

#### 2.2.3. Measures for stool donors with suspected SARS-CoV-2 infection

When developing COVID-19-related symptoms, donors were not allowed to donate faeces for two weeks in case of a negative nasopharyngeal SARS-CoV-2 test performed at the Municipal Health Services, or four weeks when no swab was taken. In case of a positive SARS-CoV-2 nasopharyngeal swab, a donor was not allowed to donate for eight weeks [19]. Eight weeks after the start of developing COVID-19 symptoms, donor faeces and serum were screened for SARS-CoV-2. Additionally, all faecal suspensions donated two weeks prior development were discarded.

### 2.3. Inclusion of patients

#### 2.3.1. Patients with recurrent *Clostridioides difficile* infection

FMT treatment for patients continued during the COVID-19 pandemic. The FMTs were initially only performed with donor faeces donated before December 2020, until the above described donor measures were instated. Inclusion and follow-up of rCDI patients was performed according to standard NDFB protocol, as previously described [7]. In short, FMT requests for rCDI from different hospitals throughout the Netherlands were evaluated by the NDFB FMT-expert panel assessing patient eligibility and providing advice on diagnosis and follow-up. Patient follow-up was routinely performed by a short questionnaire assessing clinical information at approximately three weeks, two to three months, and six months after FMT. Information about hospital admission, recurrence, antibiotic use, infections and possible FMT-related adverse events was collected [7].

#### 2.3.2. Patients with other disorders

The NDFB compassionate use program provides faecal suspensions for patients that suffer from diseases for which there is no viable alternative therapy available and who could benefit from FMT based on scientific literature. After application by the treating physician, the patient’s eligibility for FMT in combination with the existing scientific literature is carefully evaluated by an independent disease specialist and the NDFB FMT-expert panel.

#### 2.3.3. Patients included in clinical trials

Patients included in three NDFB supported clinical trials: the FECBUD (NL65976.098.18), NAFTx (NCT04465032), and ENT-trial (METC 2018:133) were evaluated. The FECBUD trial included patients with active ulcerative colitis to assess effects of four sequential FMT treatments after pre-treatment with budesonide or placebo. The NAFTx trial included patients with non-alcoholic fatty liver disease to assess effects of three sequential FMT treatments with donor versus autologous faecal suspensions on disease outcomes. The ENT-trial included post-antibiotic or post-infectious irritable bowel syndrome patients to be treated with a single FMT. Patients were randomised to receive either preceding bowel lavage or no lavage to assess the effect of lavage on engraftment of donor microbiota.

### 2.4. Statistical analysis

Statistical analysis was performed using SPSS 25 software. Comparisons between numbers of donations and between numbers of FMT requests for rCDI were performed using the two-sided independent samples t-test. Comparisons of FMT eligibility proportions, cure rates, and of adverse events were performed using Pearson’s Chi-square test and Fisher’s exact test. Odds ratios with 95% confidence intervals were calculated. CDI-free survival was calculated using Kaplan-Meier survival analysis, survival curves were compared by log-rank test. Statistical significance was considered at two-sided *p*<0.05.

### 2.5. Ethics approval

This study conforms to the ethical guidelines of the 1975 Declaration of Helsinki as reflected in a priori approval by the institution’s Human Research Committee, and was approved on December 16^th^ 2015 by the medical ethical committee of the Leiden University Medical Center (P15.154). Written informed consent was provided by donors and patients for collection and analysis of clinical data and faeces samples.

## 3. Results

### 3.1. SARS-CoV-2 incidence in stool donors

During the study period, two out of ten donors (20%) developed mild COVID-19 symptoms and tested positive for SARS-CoV-2 at the Municipal Health Services. The first donor was not allowed to donate until full rescreening eight weeks after onset of symptoms was performed and faeces was tested negative for SARS-CoV-2. No SARS-CoV-2 was detected in faeces six and ten weeks post COVID-19 symptom onset, and anti-SARS-CoV-2 IgG was detected at nine, but not at 28 weeks post symptom onset. The second donor developed COVID-19 while being temporarily inactive as a donor. The donor tested negative for SARS-CoV-2 in faeces three months after symptom onset, and remained inactive during the rest of the study period. Out of eight remaining active donors, six donors experienced COVID-19 related symptoms and requested a COVID-19 test once or multiple times at the Municipal Health Services, all with negative results. None of these eight donors showed anti-SARS-CoV-2 sero-response at any time during screening. All 46 tested donor faeces were deemed SARS-CoV-2 PCR negative (S1 Table).

### 3.2. Donations made by stool donors

**Fig 1:**
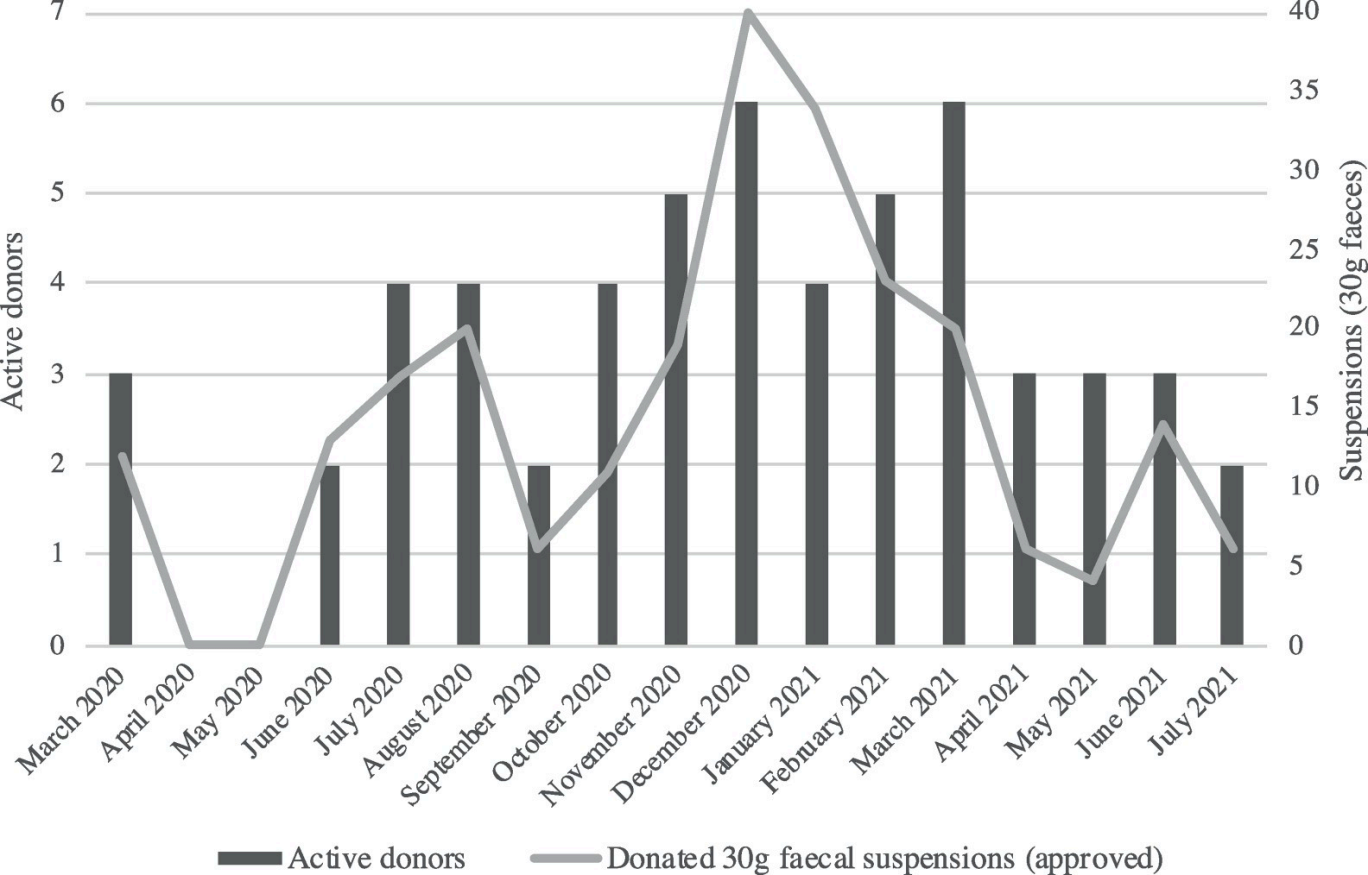
Faeces donations by donors active during the COVID-19 pandemic. The number of active NDFB donors per month, combined with the number of approved 30g faecal suspensions donated per month in the period from March 2020 to August 2021. The NDFB halted donor activities from March to June 2020.

The absolute number of approved 99ml suspensions containing 30g faeces donated between March 2020 and August 2021 was 245 (Table 1 and Fig 1). With 245 (30 gram) suspensions, 122 rCDI patients can be treated with FMT. When including the temporary halt of donor activities in 2020, a trend towards a lower average monthly number of approved 30g faecal suspensions donated during the COVID-19 pandemic was observed, compared to the four years before onset of COVID-19 (Table 1).

**Table 1:** Faeces donations (30 grams) during versus before the COVID-19 pandemic.

	Period	Donations (30g)	Months	Average/month Mean; SD	p values (two sided)^a^ p; (t-test statistic_df_)
**Pre-COVID-19**	2016–2019	1000	46	21.7 ± 13.9	
**During COVID-19**	March 2020-Aug 2021^b^	245	17	14.4 ± 11.1	*p* 0.06 (*t*_61_ 1.9)
	June 2020-Aug 2021^c^	233	14	16.6 ± 10.6	*p* 0.2 (*t*_58_ 1.3)

SD, standard deviation; df, degrees of freedom.

^a^ *p* values were calculated using independent samples t-tests. Donor donation data obtained between March 2020 and August 2021 was compared with pre-COVID-19 data (2016–2019) [7].

^b^ Period including the halt in donor activities (March 2020-June 2020).

^c^ Period excluding the halt in donor activities (March 2020-June 2020).

### 3.3. Stool donor engagement

After restarting donor activities, three donors indicated difficulty or reluctance to donate at the NDFB due to mandatory working from home, or fear of becoming infected with SARS-CoV-2 in public transport or the hospital. In December 2020, a courier was employed to collect faeces at the donors’ home or work address, resulting in re-engagement of two active donors (Fig 1). Furthermore, a new donor was included in February 2021. The NDFB did not experience shortage of available screened faecal suspensions. At the end of the study period, eight out of ten active donors reported to be fully vaccinated against SARS-CoV-2.

### 3.4. Faecal microbiota transplantation for recurrent *Clostridioides difficile* infection

#### 3.4.1. Requests for faecal microbiota transplantation

From March 2020 to August 2021, the NDFB received 67 FMT requests for treatment of rCDI. The monthly number of FMT requests during the COVID-19 pandemic (March 2020-August 2021) was similar compared to before the pandemic (2016–2019) (Table 2) [7]. During the first lockdown period (March-June 2020), when the NDFB halted all donor activities, a trend towards a lower number of FMT-requests was observed compared to previous years (2016–2019) (Table 2) [7]. Overall, no major effect of the COVID-19 pandemic on the number of FMTs requested was noted, despite a short-lived setback in requests between March and June 2020.

**Table 2:** FMT requests for rCDI during versus before the COVID-19 pandemic.

	Period	Requests	Months	Average/month Mean; SD	*p* values (two sided)^a^ *p*; (t-test statistic_df_)
**Pre-COVID-19**	2016–2019 [7]	176	41	4.3 ± 2.4	
**During COVID-19**	March 2020-August 2021	67	17	3.9 ± 1.9	*p* 0.6 (*t*_56_ 0.5)
	March 2020-June 2020	5	3	1.7 ± 1.2	*p* 0.07 (*t*_42_ 1.9)
	June 2020-August 2021	62	14	4.4 ± 1.6	*p* 0.8 (*t*_53_ 0.2)

FMT, faecal microbiota transplantation; rCDI, recurrent *Clostridioides difficile* infection; SD, standard deviation; df, degrees of freedom.

^a^ *p* values were calculated using independent samples t-tests. FMT request data obtained between March 2020 and August 2021 was compared with pre-COVID-19 data (2016–2019) [7].

#### 3.4.2. Eligibility for faecal microbiota transplantation

Between March 2020 and August 2021, 54 rCDI patients (average age 67 years, 73.6% female) were treated with FMT via nasoduodenal tube (50/54) or colonoscopy (4/54) with faecal suspensions (60 gram per treatment) prepared in 2018, 2019 and 2020 (four in January 2020, one in July 2020). Out of 67 requests, 54 patients (80.6%) were deemed eligible by the FMT expert group. This was not different from previous years (2016–2019) (Table 3) [7].

**Table 3:** Eligibility for FMT and cure rates of FMT for rCDI during versus before the COVID-19 pandemic.

		Period/timepoint	Outcome %; N	Statistics^a^ OR; [95% CI]; *p* (two sided)
**Eligibility for FMT (rCDI)**	**Pre-COVID-19 [7]**	2016–2019	73.3% (129/176)	
	**During COVID-19**	March 2020-Aug 2021	80.6% (54/67)	OR 1.51 [0.76–3.02], *p* 0.2
**rCDI cure rates**	**Pre-COVID-19 [7]**	Three weeks after FMT	91.4% (117/128)	
	**During COVID-19**	Three weeks after FMT	97.6% (41/42)	OR 0.26 [0.03–2.07], *p* 0.2
	**Pre-COVID-19 [7]**	Two months after FMT	89.2% (107/120)	
	**During COVID-19**	Two months after FMT	90.3% (28/31)	OR 0.88 [0.24–3.31], *p* 0.9
	**Pre-COVID-19 [7]**	Long term follow up (median 42 weeks)	72.6% (61/84)	
	**During COVID-19**	Six months after FMT	70.0% (14/20)	OR 1.14 [0.39–3.31], *p* 0.8

FMT, faecal microbiota transplantation; rCDI, recurrent *Clostridioides difficile* infection; OR, odds ratio; 95% CI, 95% confidence interval.

^a^ *p* values were calculated using Chi Square and Fishers Exact tests. FMT eligibility and patient data obtained between March 2020 and August 2021 was compared with pre-COVID-19 data (2016–2019) [7].

#### 3.4.3. Outcome of faecal microbiota transplantation

**Fig 2:**
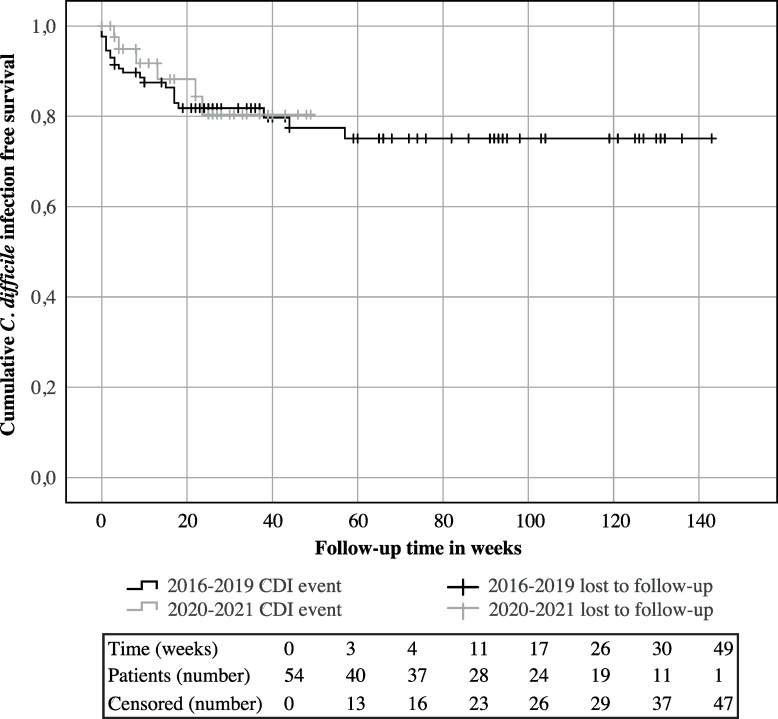
*Clostridioides difficile* infection-free survival during versus before the COVID-19 pandemic. Kaplan Meier curve of *Clostridioides difficile* infection-free survival pre (2016–2019) and during the COVID-19 pandemic (March 2020-August 2021) after treatment with faecal microbiota transplantation using faecal suspensions provided by the NDFB [7].

Follow-up was available for respectively 42 patients at three weeks (median 4 weeks, range 1–26), 31 patients at two to three months (median 17 weeks, range 9–31) and 20 patients at six months after FMT (median 31 weeks, range 25–46). The primary cure rate at three weeks after FMT was 97.6% (41/42), cure rate at two months after FMT was 90.3% (28/31), and long-term cure at six months was 70.0% (14/20). These were similar to cure rates obtained before the COVID-19 pandemic (Table 3) [7]. Furthermore, no differences were observed in CDI-free survival after FMT compared to previous years (two-sided *p* = 0.7) (Fig 2) [7]. Three patients suffered from an early relapse within two months after FMT, of which two were successfully treated with respectively fidaxomicin and vancomycin. The third patient was treated with fidaxomicin, but suffered from a CDI reoccurrence at five and eight months after FMT, for which ultimately, a second FMT was required. Three additional patients suffered from a CDI reoccurrence, respectively three, five, and five and a half months after FMT. One patient had a history of ulcerative colitis, the other patients had received antibiotics prior to the CDI reoccurrence, respectively amoxicillin-clavulanic acid and floxacillin.

#### 3.4.4. Safety of faecal microbiota transplantation

Similar to before the COVID-19 pandemic, no FMT related or probably FMT related serious adverse events were reported (S2 Table) [7]. One patient reported a COVID-19 infection three weeks after FMT (2.4%, 1/42). The FMT was performed with donor faeces donated in 2018, ruling out the possible transfer of SARS-CoV-2 from donor to patient. Five patients were hospitalised within three weeks after FMT (11.9%, 5/42), of which four with possibly FMT related complications (9.5%, 4/42) (S2 Table). No difference was observed between possibly FMT related adverse events within three weeks during versus before the COVID-19 pandemic (9.5% versus 7.8%) (OR = 1.24, 95%CI [0.36–4.26], two-sided *p* = 0.7) [7].

#### 3.4.5. Treatment delay

FMT treatment was delayed for two patients due to COVID-19 related causes. For the first patient, the FMT procedure was postponed four weeks due to the patient’s fear of becoming infected with SARS-CoV-2 within the hospital. Oral vancomycin treatment (4dd 250mg) was continued until the FMT. For the second patient, FMT was postponed due to a known COVID-19 case within the patient’s household, upon which the patient developed COVID-19 with mild symptoms. FMT was postponed for three weeks, until the quarantine period was ended. Vancomycin treatment (4dd 250mg) was continued until FMT.

### 3.5. Faecal microbiota transplantation for other indications

From March 2020 to August 2021, eight patients (0.5/month) were treated with 21 FMTs (1.2/month) for disorders other than rCDI, facilitated by the NDFB compassionate use program. Six out of eight patients were treated with multiple FMTs (range 2–4). In 2019, five patients (0.4/month) were treated with five FMTs (0.4/month) within the program. Therefore, no effect of the COVID-19 pandemic on continuation of the compassionate use program was observed.

### 3.6. Faecal microbiota transplantation in clinical trials

Clinical studies performed within the Leiden University Medical Center were halted between mid-March and June 2020. The FECBUD trial started in May 2019 and was concluded in December 2020. No new patients were included between mid-March and June 2020, leading to a delay in patient inclusion of two and a half months. In total, 24 patients were treated (92 FMTs), the trial was concluded without further COVID-19 related exclusions or delays. The NAFTx trial started in January 2020 but was halted between March and August 2020 resulting in a five month delay in patient inclusion and treatment. Up to August 2021, 20 out of 21 patients were treated (56 FMTs). The prolonged delay was caused, among other reasons, due to the heavy burden of COVID-19 on the radiology department, which reduced the capacity for magnetic resonance imaging (MRI) scans upon multiple NAFTx trial related hospital visits. Furthermore, the medical ethical committee imposed multiple COVID-19 related restrictions to minimise the for this study required patient transfer between hospital departments. The ENT-trial (Amsterdam UMC) started in 2019, but was halted from March 2020 until further notice. A total of 12 patients were treated with a single FMT up to the imposed COVID-19 restrictions. Reopening the study has proven to be difficult due to the limited availability of personnel to perform endoscopic procedures such as FMT.

## 4. Discussion

FMT is an effective and safe treatment for rCDI patients and should for these patients be considered a non-postponable treatment [19]. Drawbacks of FMT are its unstandardised nature and the theoretical possibility to co-transplant undesired pathogens such as SARS-CoV-2. In order to prevent transmission from donor to recipient, stool banks have suspended all donor activities in the beginning of the COVID-19 pandemic, some only issuing donor suspensions prepared in the pre-COVID-19 era. In this study, we show that with quick adaptations in workflow and appropriate additional donor screening, effective and efficient stool banking to facilitate FMT treatment is possible during the COVID-19 pandemic.

We observed minor effects of the pandemic on stool donation and requests for FMT treatment. Although only a minority of donors (20%) developed COVID-19, a downward trend in the average number of NDFB donor donations was observed compared to the pre-COVID-19 period. We can however not exclude that this was part of a normal yearly fluctuation pattern in the number of donations, due to the relatively short period of data collection. Despite a small temporary setback in FMT requests for rCDI during the first lockdown, the overall number of FMTs requested for rCDI, eligibility for FMT, cure rate of rCDI, CDI-free survival after FMT, and the number of possibly FMT related adverse events within three weeks after FMT were similar to before the emergence of SARS-CoV-2. This reflects FMT is considered, in principle, an essential and non-postponable treatment for rCDI. Clinical research trials were hampered by the COVID-19 pandemic and two out of three resumed research activities after taking adequate security measures. Results of the NDFB are in line with a study published by the Italian stool bank, which reported the possibility to maintain standard volumes, efficacy and safety of FMT during COVID-19, by adopting specific changes in the operational FMT centre workflow [15]. Furthermore, a United Kingdom (UK)-based stool bank resumed donor screening and FMT services after an initial halt, and also United States (US) based stool banks have reported that FMT can safely be performed during the COVID-19 pandemic with appropriate donor screening [14, 20]. Finally, resumption of activities was indicated by stool banks from Germany and Denmark in personal communication with the NDFB.

Although all stool banks adjusted their workflow due to COVID-19, differences existed in methods used for SARS-CoV-2 donor screening. Ianiro and colleagues (Italy) described detection of SARS-CoV-2 by PCR via nasopharyngeal swab and serology (IgM + IgG) upon every donation [15]. Quraishi and colleagues (UK) reported to perform faecal and nasopharyngeal SARS-CoV-2 PCR screening before and after periods of ten consecutive donation days [14]. Khanna and colleagues (US) reported to screen donors by nasopharyngeal swab PCR and serology (IgG) every two weeks, and described donor temperature logging in one of the stool banks [20]. The NDFB implemented additional SARS-CoV-2 screening in faeces and SARS-CoV-2 antibody screening in serum to the two-to-three-monthly regular bookend donor screening, hereby limiting screening frequency and intensity. Of note, the NDFB protocol did not rely on nasopharyngeal swabs, further reducing the burden for donors. This strategy differs from the previously published strategies and the international consensus statement [19]. Given the widespread expectations that COVID-19 will not disappear, it is of importance that COVID-19 screening is effectively and permanently incorporated in donor screening protocols, taking the invasiveness of testing protocols, the costs of testing, and transmission risk into account. Future observations will have to clarify what is the most accurate screening regimen.

Evidence for the use of PCR tests for detection of SARS-CoV-2 RNA in stool is growing. Manzoor and colleagues described similar, adequate detection of real-time PCR stool assays in faecal samples and FMT suspensions spiked with SARS-CoV-2 inactivated lysate [22]. Additionally, three validation studies described the validation and optimisation of a PCR assay for SARS-CoV-2 detection in faecal material, showing adequate detection in faeces in spike-in experiments [23–25]. Finally, Natarajan and colleagues described optimised protocols for detection of SARS-CoV-2 in stool, using PCR [26]. However, determining clinical validity and sensitivity of SARS-CoV-2 tests in stool remains a challenge. While evidence indicates that SARS-CoV-2 RNA can be present in stool for a prolonged time after infection and that SARS-CoV-2 may potentially be able to infect the gut, only few studies with limited sample size showed the presence of intact, infectious virus in stool, in all cases from hospitalised, symptomatic COVID-19 patients, or samples with high viral copy numbers [12, 27–33]. Multiple studies failed to detect viable SARS-CoV-2 in RNA positive stool, and the presence of SARS-CoV-2 RNA in faeces may therefore not necessarily indicate the presence of viable, infectious SARS-CoV-2 [12, 34].

The present study has limitations. Due to the limited number of patients and donors included in the analyses, results should be interpreted with caution. Though the two-to-three-month quarantine period between bookend screenings was indicated adequate for detection of a wide range of viral and bacterial pathogens and multi-drug resistant organisms (MDROs) in the past, in theory, asymptomatic SARS-CoV-2 positive donors with not yet an, or low detectable sero-response could remain unnoticed, due to false negative serologic test outcome [7, 35]. Furthermore, five patients in this study were treated with FMT suspensions donated after 2019, of which only one later than March 2020, due to sufficient supplies and a ‘*first in first out’* approach with a shelf life period of two years [7]. Finally, only one actively donating stool donor was reported COVID-19 positive. Therefore, a definite analysis of unexpected undetected SARS-CoV-2 in donors, and unexpected transfer from negative donors to patients could not be performed.

Although our sample size is limited, the data in the present report indicate the possibility of efficient and effective stool banking during the COVID-19 pandemic, and the continuous need for FMT suspensions. Sufficient stock of quality assured faecal suspensions by stool banks is essential, as potential outbreaks of disease in the future may again force stool banks to halt all donor activities. The quick adaptations needed in response to the COVID-19 pandemic underline the benefit of centralised stool banks or FMT expert centres capable of quickly updating donor screening and manufacturing procedures, quality control and careful and long-term monitoring of outcomes and adverse events. Setting up a national or even international registry for both donor and patient follow-up would move FMT as quality-assured treatment to the next level.

In conclusion, FMT as treatment with the aim to restore a patient’s perturbed microbiota remains the standard therapy for patients with multiple rCDI and is a non-postponable treatment during COVID-19. By not relying on nasopharyngeal swab PCR, additional measures to minimise the risk of potential transfer of SARS-CoV-2 were incorporated in existing donor screening protocols without increasing the burden for stool donors, while still allowing for effective and efficient FMT treatment during the ongoing COVID-19 pandemic. As SARS-CoV-2 appears here to stay, stool banks should evaluate and if necessary adapt SARS-CoV-2 donor screening protocols for long term sustainability, weighing in its efficacy and the burden for stool donors. Sharing the experiences of stool banks in response to SARS-CoV-2 in particular, and (new) infectious diseases in general, helps the utilisation, standardisation and maturation of stool banks worldwide, and FMT as therapy.

## Supporting information

S1 Table Donor serum and faeces screening for SARS-CoV-2. (DOCX)

S2 Table Adverse events after FMT for rCDI during the COVID-19 pandemic. (DOCX)

S3 Table Abbreviation list. (DOCX)
