# Supplementary material for: How to prepare stool banks for an appropriate response to the ongoing COVID-19 pandemic: Experiences in the Netherlands and a retrospective comparative cohort study for faecal microbiota transplantation
Source: PLoS One. 2022 Mar 17;17(3):e0265426. doi: 10.1371/journal.pone.0265426 (PMC8929558; doi:10.1371/journal.pone.0265426)
Supplement: S1 Table — (DOCX) [file pone.0265426.s001.docx]

**S1 Table. Donor serum and faeces screening for SARS-CoV-2.**

|  | **Serology (Architect/Alinity IgG, anti-SARS-CoV-2 N-protein)** | | | **PCR Faeces (E-gene)** | | |
| --- | --- | --- | --- | --- | --- | --- |
| **Donor#** | **Timepoint^a^ (weeks)** | **Index value** | **Interpretation** | **Timepoint^a^ (weeks)** | **Cq** | **Interpretation** |
| D1 | t=0 | 0.08 | Neg | t=0 | 0 | Neg |
|  | t=16 | 0.73 | Neg | t=16 | 0 | Neg |
|  |  |  |  | t=27 | 0 | Neg |
|  |  |  |  | t=35 | 0 | Neg |
|  |  |  |  | t=41 | 0 | Neg |
| D2 | t=0 | 0.05 | Neg | t=0 | 0 | Neg |
|  | t=11 | 0.08 | Neg | t=11 | 0 | Neg |
|  | t=24 | 0.09 | Neg | t=21 | 0 | Neg |
|  | t=40 | 0.06 | Neg | t=22 | 35.05 | Neg |
|  | t=58 | 0.08 | Neg | t=24 | 36.24 | Neg |
|  |  |  |  | t=25 | 41.83 | Neg |
|  |  |  |  | t=39 | 0 | Neg |
|  |  |  |  | t=54 | 0 | Neg |
| D3 | t=0 | 0.02 | Neg | t=0 | 0 | Neg |
|  | t=10 | 0.01 | Neg | t=11 | 0 | Neg |
| D4 | t=0 | 0.02 | Neg | t=0 | 0 | Neg |
|  | t=48 | 0.05 | Neg | t=11 | 0 | Neg |
|  | t=62 | 3.25 | Pos | t=15 | 0 | Neg |
|  | t=81 | 0.43 | Neg | t=18 | 0 | Neg |
|  |  |  |  | t=27 | 0 | Neg |
|  |  |  |  | t=33 | 0 | Neg |
|  |  |  |  | t=41 | 0 | Neg |
| D5 | t=0 | 0.04 | Neg | t=0 | 0 | Neg |
|  | t=14 | 0.49 | Neg | t=14 | 0 | Neg |
|  | t=28 | 0.79 | Neg | t=26 | 0 | Neg |
|  | t=40 | 0.02 | Neg | t=36 | 0 | Neg |
|  | t=53 | 0.02 | Neg |  |  |  |
| D6 | t=0 | 0.00 | Neg | t=0 | 0 | Neg |
|  | t=10 | 0.03 | Neg | t=13 | 0 | Neg |
|  |  |  |  | t=19 | 0 | Neg |
|  |  |  |  | t=28 | 0 | Neg |
|  |  |  |  | t=36 | 0 | Neg |
|  |  |  |  | t=44 | 0 | Neg |
| D7 | t=0 | 0.02 | Neg | t=0 | 0 | Neg |
|  | t=17 | 0.02 | Neg | t=25 | 0 | Neg |
|  | t=45 | 0.02 | Neg | t=29 | 0 | Neg |
|  |  |  |  | t=33 | 0 | Neg |
|  |  |  |  | t=39 | 0 | Neg |
|  |  |  |  | t=47 | 0 | Neg |
| D8 | t=0 | 0.01 | Neg | t=0 | 0 | Neg |
|  | t=9 | 0.01 | Neg | t=4 | 0 | Neg |
|  |  |  |  | t=9 | 0 | Neg |
|  |  |  |  | t=38 | 41.36 | Neg |
| D9 | t=0 | 0.18 | Neg | t=0 | 0 | Neg |
|  |  |  |  | t=51 | 0 | Neg |
| D10 | t=0 | 0.01 | Neg | t=0 | 0 | Neg |
|  |  |  |  | t=8 | 0 | Neg |

^a^ t=0: First serum/faeces screening of a donor during the study period, varied between donors.
