# Supplementary material for: How to prepare stool banks for an appropriate response to the ongoing COVID-19 pandemic: Experiences in the Netherlands and a retrospective comparative cohort study for faecal microbiota transplantation
Source: PLoS One. 2022 Mar 17;17(3):e0265426. doi: 10.1371/journal.pone.0265426 (PMC8929558; doi:10.1371/journal.pone.0265426)
Supplement: S2 Table — (DOCX) [file pone.0265426.s002.docx]

**S2 Table. Adverse events after FMT for rCDI during the COVID-19 pandemic.**

|  |  | **Adverse event^a^** | **Percentage** |
| --- | --- | --- | --- |
| **≤3 weeks after FMT** | **Possibly FMT related** | Urinary tract infection; (one patient with medical history of recurrent urinary tract infections, and one patient with renal insufficiency on dialysis) | 4.8% (2/42) |
|  |  | Hospitalised with stomach cramps and rectal blood loss. | 2.4% (1/42) |
|  |  | Hospitalised with recurrent cholangitis; patient with a medical history of primary sclerosing cholangitis | 2.4% (1/42) |
|  |  | Hospitalised with *Escherichia coli* peritonitis and bacteraemia after second FMT (direct relation with FMT excluded: patient *E.coli* was not found in donor sample. Patient *E.coli* and donor faeces were negative for enteropathogenic *E.coli^b^*) | 2.4% (1/42) |
|  |  | Hospitalised with cholecystitis and bacteraemia with multiresistent *Klebsiella* | 2.4% (1/42) |
|  | **Non-FMT related** | Hospitalized with malaise and nausea due to pleural fluid, caused by lung carcinoma progression | 2.4% (1/42) |
| **>3 weeks after FMT** | **Possibly FMT related** | 4x Urinary tract infection (indicated by patients respectively six, nine, ten and 11 months after FMT) | 20% (4/20) |
|  |  | Microscopic colitis upon colonoscopy six months after FMT, successfully treated with budesonide | 5% (1/20) |

FMT, faecal microbiota transplantation; rCDI, recurrent *Clostridioides difficile* infection.

^a^ (Severe) adverse events reported between March 2020 and August 2021 were included.
^b^ Enteropathogenicity was tested with sequencing and targeted multi-plex PCR for STEC (shiga toxin producing *Escherichia coli*), EIEC (entero-invasive *E.coli*), EAEC (entero-aggregative *E.coli*), ETEC (entero-toxic *E.coli*) and EPEC (entero-pathogenic *E.coli*).
