# Supplementary material for: How to prepare stool banks for an appropriate response to the ongoing COVID-19 pandemic: Experiences in the Netherlands and a retrospective comparative cohort study for faecal microbiota transplantation
Source: PLoS One. 2022 Mar 17;17(3):e0265426. doi: 10.1371/journal.pone.0265426 (PMC8929558; doi:10.1371/journal.pone.0265426)
Supplement: S3 Table — (DOCX) [file pone.0265426.s003.docx]

**S3 Table**

**Abbreviation list**

| **Abbreviation** | **Full name** |
| --- | --- |
| 95% CI | 95% confidence interval |
| CDI | *Clostridioides difficile* infection |
| COVID-19 | coronavirus disease 2019 |
| Df | degrees of freedom |
| DNA | deoxyribonucleic acid |
| E-gene | envelope gene |
| FDA | United States Food and Drug Administration |
| FMT | faecal microbiota transplantation |
| IgG | immunoglobulin G |
| IgM | immunoglobulin M |
| LUMC | Leiden University Medical Center |
| MDRO | multi-drug resistant organism |
| METC | medical ethical committee |
| MRI | magnetic resonance imaging |
| NDFB | Netherlands Donor Feces Bank |
| N-protein | nucleocapsid protein |
| OR | odds ratio |
| PCR | polymerase chain reaction |
| rCDI | recurrent *Clostridioides difficile* infection |
| RNA | ribonucleic acid |
| RT-PCR | reverse transcription polymerase chain reaction |
| SARS-CoV-2 | severe acute respiratory syndrome coronavirus-2 |
| SD | standard deviation |
| UK | United Kingdom |
| US | United States of America |
